# Task-Dependent Performance of Wearable Multimodal Biofeedback in Physical Rehabilitation: A Longitudinal Post-Stroke Case Study

**DOI:** 10.3390/healthcare14131823

**Published:** 2026-06-23

**Authors:** Cristiana Pinheiro, Joana Figueiredo, Tânia Pereira, Cristina Cruz, João Cerqueira, Cristina P. Santos

**Affiliations:** 1 Center for MicroElectroMechanical Systems (CMEMS), University of Minho, 4800-058 Guimarães, Portugal; joana.figueiredo@dei.uminho.pt (J.F.); cristina@dei.uminho.pt (C.P.S.); 2 LABBELS-Associate Laboratory, University of Minho, 4800-058 Guimarães, Portugal; 3 Department of Physical Medicine and Rehabilitation, Hospital of Braga, 4710-243 Braga, Portugal; tania.pereira@ulsb.min-saude.pt (T.P.); cristina@dacruz.pt (C.C.); 4 Life and Health Sciences Research Institute (ICVS), University of Minho, 4710-057 Braga, Portugal; jcerqueira@med.uminho.pt; 5 Clinical Academic Center (2CA-Braga), Hospital of Braga, 4710-243 Braga, Portugal

**Keywords:** augmented reality, center of mass, feasibility, joint angle, muscle activity

## Abstract

Background/Objectives: Wearable technology is increasingly used to provide biofeedback in physical rehabilitation; however, there is no consensus on which biofeedback parameter is most appropriate for clinical use, as most studies evaluate only one arbitrarily selected parameter. This study presents a wearable multimodal biofeedback system integrating multiple parameters selected based on the prior literature and evaluates its feasibility, usability, and implementation within a rehabilitation context through a longitudinal post-stroke case study. Methods: The system integrates inertial and electromyographic sensors to monitor centre of mass (CoM-B), joint angle (ANG-B), and muscle activity (EMG-B), delivering real-time sensory cues based on the monitored parameters. Feasibility was assessed in a post-stroke participant (male, 32 years, 29 months post-stroke, left hemiparesis, Fugl-Meyer Lower Extremity Score = 27) across 15 sessions involving stand-to-sit, split-stance weight shifting, and walking tasks. Each task was practiced with all three biofeedback parameters, with five sessions per parameter. Results: The motor performance varied across biofeedback parameters and tasks. CoM-B was associated with favourable trends in motor performance during stand-to-sit, showing improvements in medio-lateral displacement (0.03/session); ANG-B during walking, showing increased ankle dorsiflexion (1 deg/session); and EMG-B during split-stance weight shifting, showing increased tibialis anterior activation (5 µV/session). Conclusions: The findings generate the hypothesis that the ability of biofeedback to elicit favourable motor performance is task-dependent, suggesting that the choice of biofeedback parameters may need to be adapted to task demands. The system demonstrated high usability and feasibility, supporting its potential for post-stroke rehabilitation. Further studies are needed to test the generated hypothesis and evaluate the system efficacy.

## 1. Introduction

Integrating wearable technology, such as sensors and augmented reality devices, in physical rehabilitation to provide biofeedback on motor behaviour is a recent approach which complements conventional physiotherapy [1]. Wearable sensors can monitor motor behaviour in real time and inform the user through visual, auditory, or haptic cues delivered by augmented reality devices, thereby increasing behaviour awareness and encouraging self-directed motor control [1]. These biofeedback cues can contribute to accelerating the recovery process with repetitive physical training [2]. This repetitive training is fostered by wearable technology, allowing it to occur beyond specialized facilities and without the postural constraints imposed by traditional non-wearable screens [3,4].

Multiple wearable sensors have been used to monitor parameters describing motor behaviour [4], namely, pressure sensors [5], force sensors [6], and inertial measurement units [7]—monitoring biomechanical parameters—and electromyographic (EMG) and electroencephalographic sensors [8,9,10], monitoring physiological parameters. However, there is currently no consensus on the most clinically useful biofeedback parameter, and most studies evaluate a single arbitrarily selected parameter [4]. To address this limitation, we developed a novel wearable multimodal system, combining inertial and EMG sensors with augmented reality glasses, to provide biofeedback on centre of mass (CoM-B), joint angle (ANG-B), or muscle activity (EMG-B) parameters. This multimodal approach was guided by evidence showing that post-stroke biofeedback systems most commonly rely on inertial and electromyographic sensing modalities [10], and was further guided by the need to capture complementary and hierarchically distinct levels of motor control, including global postural coordination, joint-specific kinematics, and muscle-level activation. We aim to present the design of this wearable multimodal biofeedback system and evaluate its feasibility in physical rehabilitation through a longitudinal post-stroke case study.

Stroke is a leading cause of long-term disability worldwide, often resulting in decreased dynamic balance control, weight shifting, dorsiflexion, and muscle strength in comparison with healthy individuals [3,11]. Rehabilitation is essential to restore function and prevent complications, and emerging technologies such as biofeedback provide patients with real-time information to guide movement training [12]. Recent research supports the effectiveness of biofeedback for post-stroke rehabilitation. Our scoping review on biofeedback systems designed for post-stroke gait rehabilitation found evidence indicating improvements in walking speed, step length, muscle activation, and standardized clinical tests [10]. Systematic reviews report that virtual/augmented reality biofeedback approaches can yield functional gains in gait and balance when used alongside conventional physiotherapy, with some evidence of superior outcomes compared to conventional physiotherapy alone [13]. Based on this evidence, we selected a post-stroke participant as a suitable case study to investigate the feasibility of our biofeedback system and to preliminarily explore participant response to different biofeedback parameters (CoM-B, ANG-B, EMG-B) during physical rehabilitation tasks.

A single-case feasibility design was adopted due to the exploratory nature of this study and the early-stage development of the multimodal biofeedback system. In line with recommendations for early-stage rehabilitation technology research [14,15], the primary aim of this study was not hypothesis testing, comparative evaluation, or efficacy assessment, but rather feasibility assessment, including usability, operability, and preliminary exploration of participant responsiveness to different biofeedback parameters. The longitudinal approach was intentionally selected to evaluate within-subject adaptation across 15 rehabilitation sessions, reflecting the temporal structure of clinical post-stroke rehabilitation, which inherently relies on iterative training over time rather than isolated exposures. In contrast to cross-sectional designs, which provide only a static snapshot of performance, the present design enabled the capture of time-dependent dimensions of feasibility, including motor adaptation trends, usability, and perceived workload across repeated use. This is particularly relevant in post-stroke populations, where substantial inter-individual variability can obscure meaningful within-subject changes when using cross-sectional comparisons.

## 2. Materials and Methods

### 2.1. Wearable Multimodal Technology

**Figure 1:**
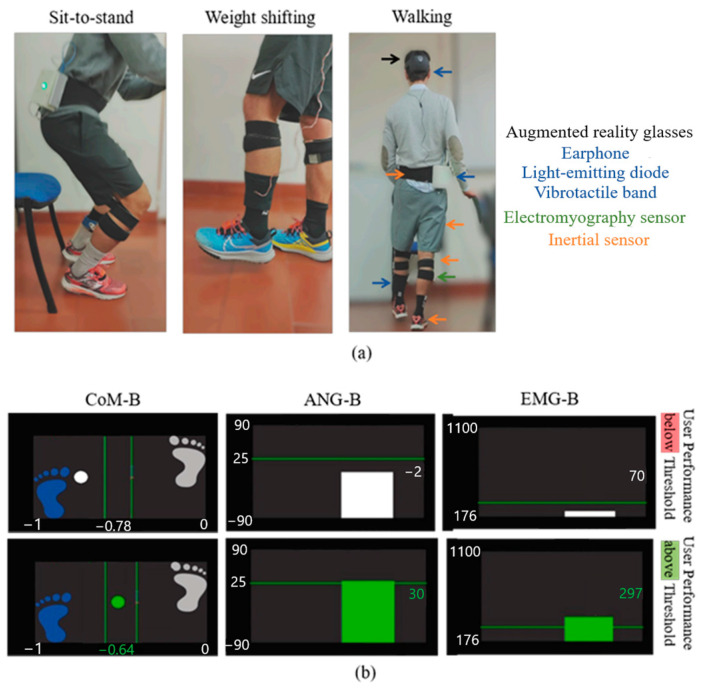
(**a**) Participant performing stand-to-sit, split-stance weight shifting, and walking tasks guided by the wearable multimodal biofeedback and (**b**) visual cue overview for CoM-B, ANG-B, and EMGB parameters.

The wearable multimodal technology used to provide biofeedback (Figure 1a) integrated multiple sensing modalities. Multimodality in this context refers to the use of different sensor types based on the literature to capture complementary aspects of motor behaviour, including inertial measurement units (Awinda system, Xsens, Netherlands) to quantify biomechanical parameters—joint angle and medio-lateral CoM position—and electromyographic sensors (Trigno system, Delsys, Natick, MA, USA) to measure physiological parameters, specifically muscle activity. The joint angle and muscle activity match biomechanical and physiological single-joint biofeedback parameters, as they primarily reflect the mechanical and muscular response of one joint in isolation, while the medio-lateral CoM position works as a multi-joint biofeedback parameter, as it represents the global outcome of coordinated movements across multiple joints, including the ankle, knee, hip, and trunk, reflecting overall balance and weight distribution [16].

The system delivered multiple sensory cues to accommodate post-stroke participants with varying sensory deficits. Namely, it included visual cues via augmented reality glasses (HoloLens 2, Microsoft Corporation, Redmond, WA, USA), and auditory and vibrotactile cues via an instrumented textile band. The sensory cues were controlled in real time according to the measured parameters.

### 2.2. Biofeedback

The biofeedback was continuously provided as a virtual circle/bar that moves/increases in proportion to the measured parameter (Figure 1b). In addition, the colour of the virtual circle/bar changed from white to green if the participant reached a virtual threshold line, indicating favourable motor response (positive reinforcement). The threshold line was adapted in real time based on the participant’s immediately preceding executions (Figure S1), allowing the system to maintain a level of challenge that was tailored to the user’s current motor performance.

Vibrotactile cues in the paretic lower limb and auditory cues were provided simultaneously with the green cues. Auditory feedback was delivered through a single in-ear earphone. Two sound configurations were used depending on duration of the feedback: for short feedback (all tasks and parameters except CoM-B during stand-to-sit), a continuous 200 Hz pure tone was applied, while for longer feedback, a piano-like melodic tone was used instead to improve listening comfort. Sound intensity was calibrated to ensure that verbal instructions from the supervising researcher remained clearly audible during sessions. Vibrotactile feedback was delivered via an elastic textile band positioned around the paretic lower limb, providing a continuous 200 Hz vibration at 1.8 g amplitude.

The visual cues from the augmented reality glasses were deactivated (feedback fading) to challenge the patient, when the threshold was reached for consecutive periods, and reactivated to avoid frustration, when the threshold was not consecutively reached. In this way, biofeedback was personalized according to the patient’s imminent performance, allowing for challenging but not frustrating physical training. This strategy aims to reduce visual attentional demand while sustaining multisensory reinforcement through less cognitively demanding cues (vibrotactile and auditory) [17].

Operability tests were conducted before this study to verify the system’s performance. The actuator CPU operated at a frame rate of 50–60 Hz, and the sensor packet drop rate remained below 0.02%. The mean inter-packet delay was 24 milliseconds with a maximum observed delay of 979 milliseconds. Prior work has demonstrated the successful validation of the proposed multimodal biofeedback in a cohort of healthy participants [16].

### 2.3. Post-Stroke Participant

The experimental protocol was conducted at the University of Minho following the Declaration of Helsinki and approval by the Ethics Committee CEICVS 006/2020. A post-stroke participant gave informed consent for inclusion before participating in this study. He was a 32-year-old male participant (body mass: 68 kg, height: 1.70 m) who possessed a higher education level, used technology daily, and practiced exercise every day before stroke occurrence. An ischemic stroke occurred 29 months before inclusion in this study, affecting his body’s left side (Fugl-Meyer lower-limb motor subscale = 27). He was able to understand and follow the experimental instructions, was able to complete the 10 m walk test without the use of a walking aid, did not present other neurological conditions or diseases that interfered with the ability to walk or receive biofeedback, and did not have any untreated medical conditions or spasticity that had not been adequately treated.

### 2.4. Biofeedback Parameters and Favourable Motor Performance

Post-stroke motor impairments in the present participant included reduced ankle dorsiflexion, likely associated with diminished tibialis anterior activation and relative overactivity of the plantar flexor muscles [18,19,20]. These deficits informed the selection of biofeedback parameters used throughout the intervention, as follows.

The sagittal ankle angle (ANG-B) and tibialis anterior muscle activity (EMG-B) were included as biomechanical and physiological single-joint parameters, respectively, providing localized information on ankle range of motion and tibialis anterior activity. These parameters were specifically targeted to address the participant’s impaired dorsiflexion control. In contrast, the medio-lateral centre of mass position (CoM-B) was selected as a multi-joint parameter, reflecting whole-body coordination and dynamic balance.

Favourable motor performance was defined in a task-specific manner for this participant. During non-locomotor tasks, favourable motor performance was characterized by increased tibialis anterior activation and ankle dorsiflexion, together with reduced medio-lateral CoM displacement, indicating enhanced balance control. During locomotor tasks, favourable performance included increased medio-lateral CoM displacement, reflecting improved weight transfer.

### 2.5. Physical Rehabilitation Complemented by Biofeedback

A longitudinal protocol including 18 sessions was implemented to evaluate the feasibility of the system in physical rehabilitation (Figure S2). It comprised a pre-biofeedback session, 15 biofeedback sessions (2 sessions per week for 8 weeks), a post-biofeedback session, and a follow-up session (1 month after the last biofeedback session). During the biofeedback sessions, the participant was instructed to perform conventional lower-limb motor tasks in physiotherapy guided by the CoM-B, ANG-B, or EMG-B biofeedback. Each biofeedback modality (CoM-B, ANG-B, EMG-B) was delivered in separate training blocks of 5 consecutive sessions, limiting the cognitive load. A random sequence of biofeedback modalities was generated before the intervention (resulting sequence: CoM-B → ANG-B → EMG-B). However, because only one participant was included, potential sequence effects could not be separated from modality-specific effects.

During this study, the participant maintained his usual daily conventional physiotherapy, encompassing 13 h per week, which did not involve biofeedback. As such, the observed changes cannot be attributed solely to the biofeedback intervention and should be interpreted as occurring within the context of ongoing standard rehabilitation.

Each biofeedback session (20 min total) included the following sequence of motor tasks: 5 min of stand-to-sit, 5 min of split-stance weight shifting, and 10 min of overground walking at a comfortable speed (Figure 1a). The motor tasks were selected in consultation with clinicians and based on clinical practice guidelines for inclusion of multiple dynamic tasks with progressive challenge [21]. The total duration of 20 min per session was selected based on evidence from the prior literature (typically ranges from 15 to 60 min per session [10]) and defined in consultation with clinicians.

A familiarization period was carried out for a maximum of 10 min during the first session with a biofeedback parameter to facilitate adaptation to the feedback and reduce initial cognitive burden. The participant confirmed that he was able to follow the biofeedback guidance to proceed.

The participant was instructed to achieve the threshold line during a particular phase of each motor task so he could cognitively relax during the remaining phases of the movement. The selected phases were defined based on their functional relevance to post-stroke motor impairments as follows: (1) the sitting phase of the stand-to-sit task requires controlled lowering of the body and dynamic balance regulation; (2) attempted dorsiflexion of the paretic limb in split-stance weight shifting with ANG-B and EMG-B targets ankle control deficits, and plantar flexion of the non-paretic limb with CoM-B promotes effective weight transfer; (3) the paretic swing phase in walking with ANG-B and EMG-B is governed by ankle dorsiflexion, and medio-lateral displacement toward the paretic limb with CoM-B promotes effective weight transfer. In this study, a researcher with expertise in biomedical engineering supervised the sessions and provided verbal guidance.

### 2.6. Outcomes

The outcomes were organized into three domains: first, sensor-based outcomes including sagittal ankle angle, tibialis anterior muscle activity, and medio-lateral CoM displacement; second, user-reported measures, including the NASA Task Load Index (NASA-TLX), the System Usability Scale (SUS), and participant’s open commentaries to assess the participant’s perceived workload (including cognitive impact) and overall usability of the system; finally, clinical outcomes including the 10-Meter Walk Test, Timed Up and Go, and the lower-limb motor and sensory subscales of the Fugl-Meyer Assessment to contextualize functional ability.

For the biofeedback sessions, sensor-based data were acquired at the beginning of each session by performing 1 min of each motor task. Data were time-normalized to the task cycle (0–100%). Mean and standard deviation curves across the task cycle were computed for sagittal ankle angle (deg), medio-lateral CoM displacement (normalized between 0 and 1 by feet distance), and tibialis anterior muscle activity (µV). For each session and motor task, average profiles of these variables were calculated, and the maximum averaged value was extracted during the specific phase in which the participant was instructed to reach the biofeedback threshold.

Given the single-case design, regression analyses were used for descriptive trend analyses rather than inferential statistical testing. A linear regression was applied to each sensor-based outcome across sessions, separately for each motor task and biofeedback modality. The slope of the regression quantified the direction and rate of change across sessions with the same modality and motor task. The coefficient of determination (R^2^) was used to assess the consistency of the performance across sessions, with higher values indicating more linear motor performance. Then, we compared the rate of change and linear consistency of motor performance between biofeedback modalities and motor tasks.

Favourable changes in motor performance were defined according to task demands. In stand-to-sit, favourable changes were characterized by reduced medio-lateral CoM displacement (suggesting enhanced balance control), alongside increased ankle dorsiflexion and tibialis anterior activation (supporting controlled forward tibial progression). In split-stance weight shifting and walking tasks, favourable changes were characterized by increased medio-lateral CoM displacement (reflecting improved weight transfer), as well as increased dorsiflexion and muscle activation.

NASA-TLX scores were averaged across sessions with the same biofeedback parameter to compare perceived workload. The SUS was administered during the final training session to evaluate overall system usability.

Clinical outcomes were assessed at pre-biofeedback, post-biofeedback, and follow-up sessions. For the 10-Meter Walk Test and Timed Up and Go, mean and standard deviation of execution times were calculated across three trials. For the Fugl-Meyer Assessment, total scores for the lower-limb motor and sensory subscales were determined.

A detailed description of data acquisition and processing is provided in the Supplementary Materials (Sections S1 and S2).

## 3. Results

Table 1 demonstrates the rate of change and linear consistency of motor performance across biofeedback sessions (regression across sessions in Figure S3 of Supplementary Materials).

Assessments with CoM-B revealed a decrease in medio-lateral displacement (−0.03/session) and an increase in tibialis contraction (11 µV/session) during stand-to-sit; an increase in tibialis contraction (3 µV/session) during split-stance weight shifting; and a decrease in tibialis activity (−6 µV/session) during walking.

Assessments with ANG-B showed an increase in ankle dorsiflexion (1 deg/session) during stand-to-sit, in medio-lateral displacement (0.05/session) during split-stance weight shifting, and in ankle dorsiflexion (1 deg/session) during walking.

Assessments with EMG-B showed a decrease in medio-lateral displacement (−0.02/session) during the stand-to-sit task, and an increase in tibialis contraction (5 µV/session) during split-stance weight shifting. No linear change was observed during walking.

The user-reported outcomes provided complementary information to the sensor-based trends. Table 2 shows the answers to the NASA Task Load Index regarding the training with each biofeedback parameter. None of the sessions were rated with fast rhythm and none delivered feelings of insecurity, boredom, and stress. CoM-B was perceived as requiring lower mental and physical effort, whereas EMG-B was associated with the highest perceived workload but also the highest perceived success. The participant reported increased awareness of tibialis anterior activation during EMG-B training and identified stand-to-sit as the most challenging task during CoM-B training. These observations provide contextual information regarding task difficulty, participant experience, and engagement across biofeedback modalities.

A usability SUS score of 87 (excellent) was obtained (Figure S4 from Supplementary Materials) following [22]. The lowest-rated answers were related to the doubt of people with limited educational or cognitive backgrounds not being able to benefit from biofeedback. Despite this, the participant showed willingness to use the system, was confident using it on his own, and considered it easy to use. Moreover, the participant commented that visual cues from the augmented reality glasses were very important because they helped him to achieve the threshold most of the time when the cues appeared. Also, the glasses did not produce any discomfort. Although the participant already felt very aware of his body, he reported that the biofeedback helped him gain even more functional awareness, especially regarding the tibialis muscle.

No adverse events were reported by the participant or noted by researchers. However, the split-stance weight shifting task was modified during ANG-B training by elevating the foot off the ground. This adjustment was introduced to increase the available ankle range of motion and ensure meaningful modulation of sagittal ankle angle, as the original configuration resulted in limited dorsiflexion variability and thus reduced sensitivity of the ANG-B signal.

Table 3 displays the 10 m Walk Test, Timed Up and Go, and Fugl-Meyer clinical scores at pre- and post-biofeedback, and follow-up sessions. There was a decrease in timing for the 10 m Walk Test (−3 s) and Timed Up and Go test (−1 s). The Fugl-Meyer motor (+2 score) and sensation scores increased (+1 score) after intervention and were retained in follow-up. The improvement in Fugl-Meyer motor score occurred regarding dorsiflexion of the ankle while lying down (subscale EII) and standing (subscale EIV) procedures.

## 4. Discussion

This work presents a wearable multimodal biofeedback system integrating inertial and EMG sensors to monitor CoM, joint angles, and muscle activity. Its feasibility for physical rehabilitation was assessed through a longitudinal post-stroke case study, evaluating motor performance, clinical outcomes, workload, and usability across 15 training sessions. Real-time biofeedback provides immediate information regarding movement execution, enabling the participant to compare actual motor output with the desired movement target and implement corrective adjustments during task performance [23]. Such augmented feedback may compensate for impaired intrinsic sensory processing by externally reinforcing movement-related information that would otherwise be difficult to perceive [23].

Distinct responses were observed across biofeedback parameters and motor tasks. However, further investigation in larger cohorts is required before drawing conclusions regarding parameter selection or task suitability. CoM-B was associated with reduced medio-lateral displacement and concurrent increased tibialis anterior activity during stand-to-sit. In contrast, during split-stance weight shifting, CoM-B showed limited changes, which was also reflected in participant feedback indicating that the task was insufficiently challenging for his current functional level. During walking, CoM-B was associated with increased medio-lateral displacement, while no consistent changes were observed in dorsiflexion or muscle activity. Force-based feedback has also been associated with improvements in weight distribution [24], yet this remain a mechanically constrained representation that does not capture muscular activation.

ANG-B was associated with changes in ankle dorsiflexion during stand-to-sit and walking. However, these changes were not accompanied by changes in muscle activity or medio-lateral displacement during stand-to-sit, consistent with the participant’s reported lack of perceived change in effort. Previous work on kinematic biofeedback during gait rehabilitation has demonstrated improvements in joint angle outcomes in post-stroke populations [25,26], but has largely focused on single-domain kinematic metrics without integrating physiological or whole-body measures. During split-stance weight shifting, dorsiflexion did not change, potentially reflecting the participant’s inability to generate sufficient ankle motion [8].

EMG-B was associated with increased tibialis anterior activity during split-stance weight shifting and walking, accompanied by the participant’s reported awareness of tibialis anterior activation. However, it did not translate into increased dorsiflexion possibly due to the present overactivity of the plantar flexor muscles. During stand-to-sit, muscle activation decreased unexpectedly, which may reflect the reduced intuitiveness of controlling the tibialis anterior during closed-chain dorsiflexion (tibia moves toward the foot) in contrast with open-chain dorsiflexion (foot moves towards the tibia), as reported by the participant. Prior studies investigating EMG-based biofeedback in post-stroke rehabilitation have primarily reported improvements in joint range of motion and muscle activation, often under seated conditions, and typically in populations with low spasticity [3,8].

These observations provide preliminary descriptive insights into how different biofeedback parameters may behave across motor tasks: CoM-B may be particularly relevant for tasks primarily requiring global postural control as it operates at a whole-body level, requiring integration of multiple joints and postural control mechanisms to regulate dynamic balance and weight transfer [27]; EMG-B may be particularly informative for tasks requiring targeted voluntary activation of specific musculature under constrained postural conditions as it provides direct information regarding activation of a specific muscle and may therefore facilitate selective recruitment of the tibialis anterior muscle during task execution [28]; and ANG-B may be particularly useful for locomotor tasks requiring kinematic control and timing of movement execution as it reflects the biomechanical outcome of coordinated muscle activity at the joint level, allowing the central nervous system to achieve the target movement through flexible combinations of muscle synergies [28]. However, given the single-case design and multiple confounding factors, these findings should not be interpreted as supporting clinical decision-making or modality selection guidelines. Instead, they highlight variability in responses that warrants further investigation in controlled multi-participant studies.

All biofeedback modalities were well tolerated by the participant, with no reported discomfort, insecurity, or stress. The participant reported that visual feedback was particularly helpful for achieving task goals, while multimodal feedback enhanced body awareness, notably for muscle activation.

Changes in functional outcome measures were recorded, including reductions of 3 s in the 10 m Walk Test and 1 s in the Timed Up and Go test. These changes are within the range reported in the literature (0.4–5 s for 10 m Walk Test and 0.2–2 s for Timed Up and Go) [6,8,29,30], although variability across studies was influenced by differences in feedback modality and training dose. Improvements in the Fugl-Meyer lower-limb motor score were observed in ankle dorsiflexion tasks but did not exceed the minimally clinically important difference [31], suggesting that greater training intensity or duration may be required to achieve clinically meaningful changes. These changes occurred in the context of ongoing conventional physiotherapy and sequential intervention design. Therefore, the observed improvements reflect the combined exposure to standard care and adjunctive biofeedback training.

### Limitations and Future Work

This study is limited by the single-participant design and training dose. From a clinical translation perspective, the single-participant design limits any direct generalization of the observed effects to the broader post-stroke population. An additional limitation is that sequence effects, cumulative learning effects, and the influence of concurrent conventional physiotherapy cannot be fully disentangled within the current design. Consequently, the observed differences between biofeedback modalities should be interpreted as exploratory and hypothesis-generating rather than as evidence of superior effectiveness of any specific parameter. Results may also have been influenced by daily living variations such as fatigue, sleep, or medication. Moreover, although necessary to ensure sufficient ankle kinematic modulation and a meaningful feedback signal, the modification of the split-stance weight shifting task during ANG-B training introduced variability in task execution across modalities.

Despite these limitations, the findings provide preliminary relevant insights for rehabilitation technology development. First, the results suggest that multimodal biofeedback can be successfully integrated into a real rehabilitation workflow and is well tolerated during repeated clinical sessions. Second, the observed variability in responses across biofeedback modalities and motor tasks indicates that a single feedback modality is unlikely to be optimal across all rehabilitation tasks, supporting the need for task-specific adaptation of feedback parameters. Finally, while these findings are not sufficient to inform clinical guidelines, they may assist clinicians and researchers in designing future rehabilitation protocols that consider both task demands and patient-specific motor impairments.

Future studies should employ a crossover design, include dedicated control conditions without biofeedback, and recruit larger cohorts to disentangle task-specific effects from time-dependent rehabilitation improvements. Such designs are essential to recommend optimal parameter selection by isolating the specific contribution of each biofeedback modality and minimizing bias introduced by sequential training and concurrent conventional therapy.

## 5. Conclusions

This study presented a wearable multimodal biofeedback system and evaluated its feasibility for post-stroke physical rehabilitation through a longitudinal case study. The system demonstrated high usability and successful real-time operability, supporting its feasibility for integration into rehabilitation settings.

The findings revealed task-dependent variability in motor responses across the different biofeedback parameters. These observations generate the hypothesis that the optimal biofeedback modality may need to be chosen according to task-specific motor demands.

Future work should prioritize controlled, larger-scale studies, including a crossover design with and without biofeedback periods, to isolate the specific contribution of biofeedback and establish evidence-based guidelines for clinical implementation.

## Figures and Tables

**Table 1:** Rate of change (and linear consistency, R^2^) of motor performance (sagittal ankle joint angle, tibialis anterior muscle activity, and medio-lateral CoM displacement) across biofeedback sessions with the same biofeedback parameter (CoM-B, ANG-B, or EMG-B) and motor task (STS, WS, or W). Values indicating favourable/unfavourable change were coloured in green/red. Consistent linear changes in motor performance (R^2^ ≥ 0.5) were highlighted with *.

Sensor-Based Outcome	Motor Task	Biofeedback Parameter			Favourable Change
		CoM-B	ANG-B	EMG-B	
Medio-lateral CoM displacement (0–1)	STS	−0.03 (0.75) *	0.02 (0.09)	−0.02 (0.63) *	<0
	WS	0.00 (0.01)	0.05 (0.88) *	0.00 (0.04)	>0
	W	0.06 (0.47)	0.05 (0.07)	−0.03 (0.35)	
Sagittal ankle angle (deg)	STS	1 (0.20)	1 (0.54) *	0 (0.01)	>0
	WS	2 (0.40)	0 (0.11)	−2 (0.49)	
	W	0 (0.02)	1 (0.74) *	0 (0.00)	
Tibialis anterior muscle activity (µV)	STS	11 (0.93) *	−1 (0.04)	−2 (0.06)	>0
	WS	3 (0.50) *	−1 (0.24)	5 (0.82) *	
	W	−6 (0.91) *	3 (0.45)	2 (0.29)	

STS: stand-to-sit, WS: split-stance weight shifting, W: walking.

**Table 2:** Averaged NASA Task Load Index scores considering the five sessions with CoM-B, ANG-B, and EMG-B.

NASA Task Load Index Question	NASA Task Load Index Score (1: Low to 21: High)		
	CoM-B	ANG-B	EMG-B
To what extent did you feel insecure, discouraged, irritated, stressed and bored?	0	0	0
How hard did you have to work to achieve your level of performance?	8	15	17
How successful were you in accomplishing what you were asked to do?	12	10	14
How fast was the pace of the task?	2	2	0
How physically demanding is the task?	2	11	16
How mentally demanding was the task?	5	8	12

**Table 3:** Mean and standard deviation (std) of clinical outcomes (10 m Walk Test, Timed Up and Go, Fugl-Meyer subscales for lower-limb motor function and sensation) at pre- and post-biofeedback, and follow-up sessions.

Clinical Outcomes	PRE	POS	FOLLOW UP
10 m Walk Test (s)	9.78	6.51	6.80
Timed Up and Go (s)	14.37	13.05	10.42
Fugl-Meyer motor (1–34 score)	27	29	29
Fugl-Meyer sensation (1–12 score)	11	12	12

## Data Availability

Data is unavailable due to privacy and ethical restrictions.

## Supplementary Materials

The following supporting information can be downloaded at: https://www.mdpi.com/article/10.3390/healthcare14131823/s1.

## Abbreviations

The following abbreviations are used in this manuscript:

ANG	Angle
CoM	Centre of mass
EMG	Electromyographic
