# Supplementary material for: Task-Dependent Performance of Wearable Multimodal Biofeedback in Physical Rehabilitation: A Longitudinal Post-Stroke Case Study
Source: Healthcare (Basel). 2026 Jun 23;14(13):1823. doi: 10.3390/healthcare14131823 (PMC13361856; doi:10.3390/healthcare14131823)
Supplement: Supplementary file 1 [file healthcare-14-01823-s001.zip › healthcare-4314152-supplementary.pdf]

Supplementary material

Threshold	10			10			↑ 13			13			13			↓ 5			5		
User Performance	11	16	12	15	12	**13**	20	8	12	6	8	5	12	10	**5**	6	4	10			
Visual cues	ON			OFF			OFF			OFF			ON			ON			ON		
Auditory & haptic cues	ALWAYS ON																				

Figure S1. **Schematic of automatic threshold updating.** Each square represents 15s of training during a session. Bold values highlight threshold updates. Threshold was calculated as the maximum value of the biofeedback parameter during the last 15s of training. It updated when one of the following conditions was verified: (i) the users overcame the threshold at least once every three consecutive 15s; (ii) the users did not reach the threshold during three consecutive 15s. In addition, the visual cues were deactivated when the threshold was reached for three consecutive 15s.

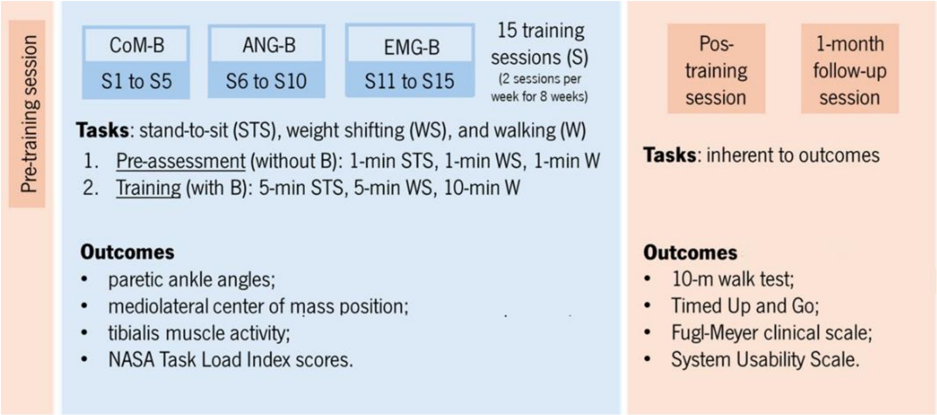

Figure S2. **Workflow of the experimental protocol with related tasks and outcomes.**

1. Data Acquisition

Assessments were carried out at the beginning of each training session by performing 1 minute of each motor task. In all session, data were monitored by commercial Xsens' inertial motion capture and Delsys' electromyographic systems to evaluate kinematic and electromyographic outcomes, respectively. Kinematic data were acquired at 100 Hz through MVN Analyze Pro software and included the medio-lateral center of mass position and paretic ankle angle in sagittal plane. Moreover, the vertical positions of the center of mass and feet, and feet orientation were acquired. Electromyographic data were acquired at 37 Hz in the form of root-mean-square through Delsys API software. Paretic muscle activation of the tibialis anterior was collected following SENIAM guidelines.

2. Data Processing

Kinematic data were automatically processed in MVN Analyze Pro software through the XKF3-hm proprietary fusion Kalman filter from Xsens. Additionally, the center of mass was post-processed to consider the current position of the right foot as the origin. Then, the positions were translated to match the orientation of the right foot while in contact with the floor. In this manner, the origin is pointing forward the subject even if he turns around in space. Moreover, it was normalized by the distance between the feet using feet positions. In this manner, normalized medio-lateral center of mass position varies between -1 and 0, coincident with left and right foot positions, respectively.

Electromyography data at 2000Hz were automatically processed by Delsys sensors with 20-450Hz filter and 100ms

window 148Hz root-mean-square. These data were received through Delsys API at 37 Hz (four samples each 27ms) and thus post-processed with a 27ms root-mean-square without overlap. Both kinematic and electromyographic data were filtered with a fourth-order Savitzky-Golay least-square polynomial filter and interpolated at 100Hz.

Movement cycles were automatically segmented using the vertical position of the center of mass for stand-to-sit (between standing positions) and non-paretic foot segment vertical position for split-stance weight shifting and walking tasks (between non-paretic foot-flats). The results of segmentation were visually inspected. Turning during walking was detected using the yaw orientation of the non-paretic foot and removed from the walking trials.

3. Results

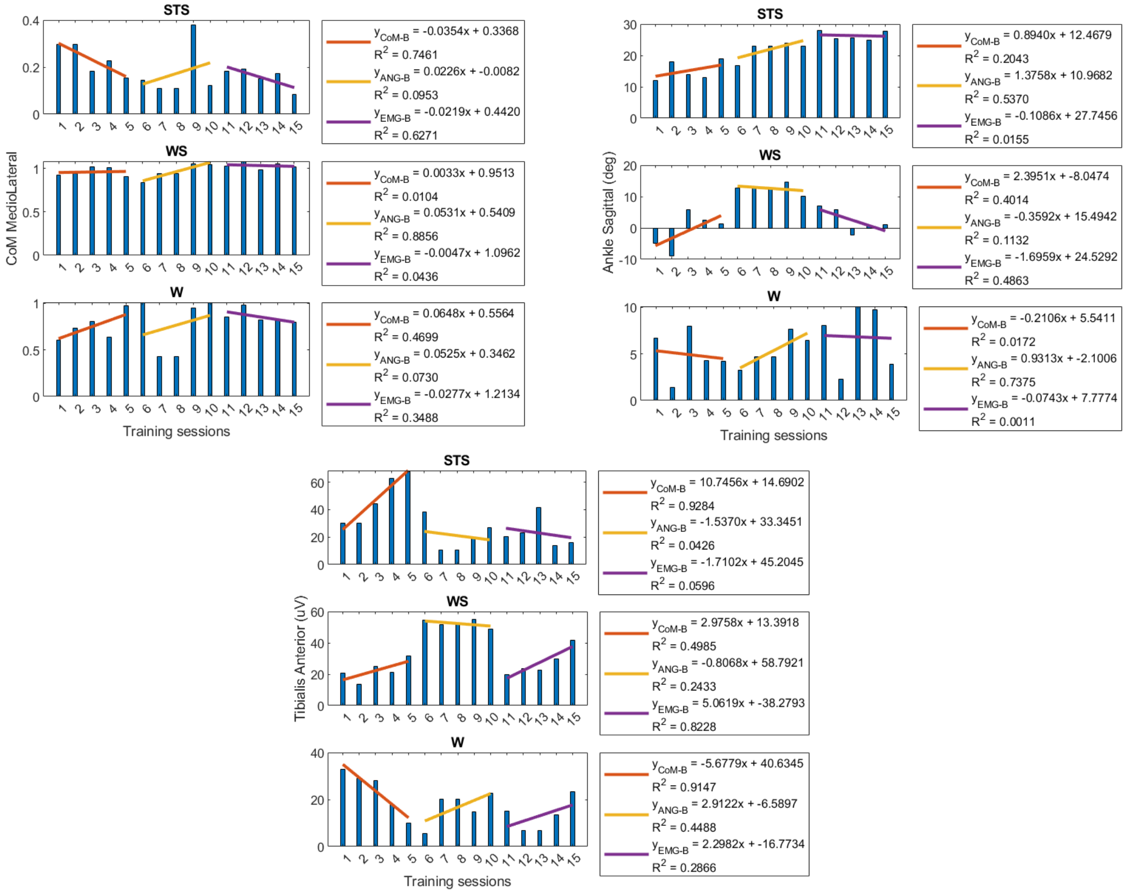

Figure S3. **Linear regression (y: outcome, x: session) of maximum averaged motor performance** (sagittal ankle angle (deg), tibialis anterior contraction (uV), and medio-lateral center of mass displacement (normalized between 0 and 1 by feet distance)) along sessions with the same of biofeedback parameter (CoM-B, ANG-B, EMG-B) and motor task (STS: stand-to-sit, WS: split-stance weight shifting, W: walking). Positive/negative sagittal ankle angles identify dorsiflexion/plantar flexion.

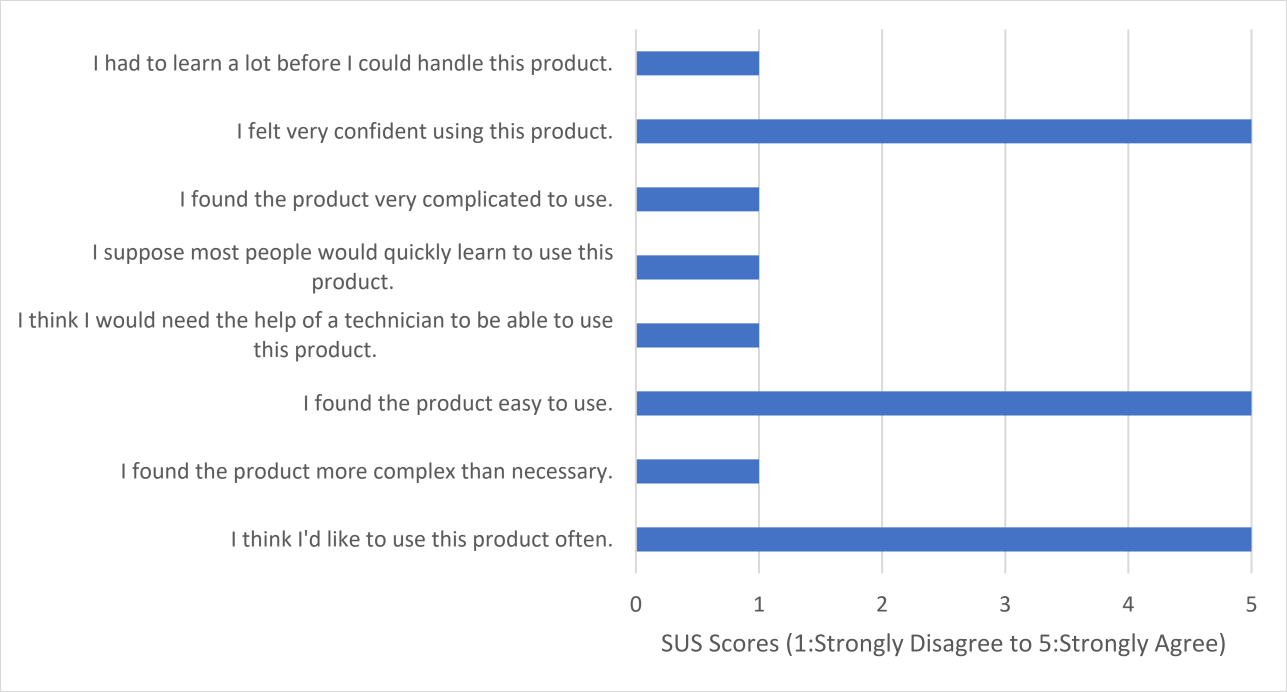

Figure S4. **System Usability Scale (SUS) scores after using the biofeedback.**
